# Formative Objective Structured Clinical Examinations (OSCEs) as an Assessment Tool in UK Undergraduate Medical Education: A Review of Its Utility

**DOI:** 10.7759/cureus.38519

**Published:** 2023-05-04

**Authors:** Khalid Al-Hashimi, Umar N Said, Taherah N Khan

**Affiliations:** 1 Vascular Surgery, Royal Shrewsbury Hospital, Shrewsbury, GBR; 2 Trauma and Orthopaedics, Huddersfield Royal Infirmary, Huddersfield, GBR; 3 General Medicine, Worcestershire Acute Hospital NHS Trust, Worcestershire, GBR

**Keywords:** objective structured clinical examination (osce), undergraduate medical education, clinical skills assessment, formative assessments, clinical competency

## Abstract

The Objective Structured Clinical Examination (OSCE) is a globally established clinical examination; it is often considered the gold standard in evaluating clinical competence within medicine and other healthcare professionals’ educations alike. The OSCE consists of a circuit of multiple stations testing a multitude of clinical competencies expected of undergraduate students at certain levels throughout training. Despite its widespread use, the evidence regarding formative renditions of the examination in medical training is highly variable; thus, its suitability as an assessment has been challenged for various reasons. Classically, Van Der Vleuten’s formula of utility has been adopted in the appraisal of assessment methods as means of testing, including the OSCE. This review aims to provide a comprehensive overview of the literature surrounding the formative use of OSCEs in undergraduate medical training, whilst specifically focusing on the constituents of the equation and means of mitigating factors that compromise its objectivity.

## Introduction and background

A paradigm shift has been seen with regard to various aspects of medical education. Alternative principles and methods have arisen over time for both the student and the teacher with respect to methods in both learning and teaching, respectively [1]. However, emphasis has also been placed on developing various modes of another component of education: assessment. Assessment and evaluation are imperative in every aspect of professional development and throughout the course of a learner’s educational process [2]. This has become increasingly important within the context of medical education due to ongoing attempts to ensure continuous patient safety [3]. Due to this, assessment processes have progressed over the years within medical education to ensure specific competencies are met by medical students, varying from multiple-choice questions to simulations [4].

Assessments are generally divided into two key sub-types: formative and summative. Both forms aim to assess a student’s competency. This is usually done by ensuring that specific learning outcomes, derived from competency-based medical education (CBME) frameworks [5], within the curricula are being met. Despite some similarities, each tends to serve different purposes [6]. Formative assessments are ones usually undertaken at various intervals throughout the course of the academic year that have the sole purpose of providing feedback to allow students to gain insight into and monitor their own progress. They additionally play a role in providing direction for teachers in altering learning activities whilst promoting more beneficial learning for areas of weakness identified following the provision of feedback [7,8]. Conversely, summative assessments generally serve as an indicator to ensure that a student has met the minimum requirement for progression to the next stage of study [2].

The Objective Structured Clinical Examination (OSCE) was initially introduced into practice in the 1970s by Ronald Harden [9]. Harden’s intentions were to improve the assessment of clinical competency resulting in the displacement of previously employed approaches; even prior to its introduction as a tool within the assessment of medical undergraduates, various shortcomings had been noted with the use of traditionally ‘long cases supplemented with smaller short cases’ [10]. The OSCE has now been described as the ‘gold standard’ for the assessment of clinical competency and holds its place as a core component within undergraduate medical examinations [11]. Due to its reliability, evidence has shown its potential to be used in both formative and summative formats [12,13].

In the Flexnerian era, medical students would typically encounter the OSCE in the later ‘clinical stages’ of their studies [14]. However, their utilisation in pre-clinical years has become more prominent [15] as medical schools within the UK have deviated away from the pre-clinical and clinical divide towards integrated approaches with early patient exposure.

Experiences with the use of formative OSCEs in the assessment of undergraduate medical students have been documented in the literature with the focus being placed on subsequent performances in summative OSCE assessments [12,16,17]. Formative OSCEs are known to have some benefits as these mock assessments have been shown to be valued by students in the past, with the added potential of improving students’ confidence in later summative assessments [18]. Despite student perceptions’ contributing to the supposed benefits of formative OSCEs, there has been some disparity as to whether they truly have any influence on performance in later summative clinical examinations. Studies have been employed by medical schools between groups of medical students to assess the influence that engagement with a formative OSCE has on their future performance in end-of-year examinations [16]. The benefits and disadvantages of formative OSCEs remain conflicting within the current literature, yet they continue to be used as a means of examining students throughout undergraduate medical training within the UK. This review aims to evaluate the evidence surrounding formative OSCEs as a mode of assessment, with a specific focus on how best to mitigate any pitfalls that may arise with their use.

The Objective Structured Clinical Examination and Miller’s pyramid

Assessment of clinical competency was initially outlined in 1990 after the introduction of a new framework by George Miller [19]. Due to initial assessment methods focusing purely on the recall of knowledge, a gap was noticed in the scope for assessing medical students in a clinical capacity. This called for the use of a more organic approach, simulating how a student was to behave in a future consultation with a patient [20]. ‘Miller’s pyramid’ was therefore established as a model that contributed to the shift away from the traditional Flexnerian approach of purely theoretical modes of assessment and towards clinical performance-based methods.

Adopting a four-tier approach, clinical competency has been subdivided into the ‘knows’, ‘knows how’, ‘shows how’ and ‘does’ categories within Miller’s pyramid. The original Flexnerian attitude to assessments within medical education would have originally placed emphasis on the cognitive components of competence [21]. Multiple-choice questions (MCQs) were used to assess knowledge as part of the ‘knows’ category, whilst ‘knows how’ involved application through extended multiple-choice questions (EMQs) or essays. The behavioural aspects of clinical competence are covered closer to the apex of the pyramid. ‘Shows’ encompasses the demonstration of learning in the format of an OSCE, whilst ‘does’ refers to a clinician’s exercising of clinical skills in daily practice usually assessed using means such as a mini-CEX or a directly observed procedure (DOPs) [20].

Placing the behavioural components of competence near the top stipulated that Miller preferred performance-based assessments as simulated environments allowed for candidates to demonstrate performance in a selected area [20]. Contrary to Miller’s beliefs, Dreyfus and Dreyfus described competence as a point within the spectrum of performance [9]; the basis for this is that assessment of ability should span beyond levels of competence to levels such as proficient and expert. Whilst the OSCE does address the level of ‘shows’, it can only be used to assess certain aspects within a medical curriculum. This is usually mitigated through the use of various assessment techniques to ensure that various aspects are assessed during the blueprinting process; by doing so, wide curricular coverage is achieved [22].

## Review

Utility equation

To ensure the provision of a rigorous procedure, various factors need to be accounted for by utilising various assessment types. The modes of assessment employed by medical schools usually include multiple approaches to address the different levels of Miller’s pyramid [1]. The use of multiple-choice examinations is typical in the assessment of a student’s cognition as it focuses on the recall of theoretical knowledge [23]. The OSCE, on the other hand, intends to provide a generalised overview of one’s approach to a clinical situation and their specific behaviours in doing so; this may vary depending on the clinical scenario. Assessment of communication skills may be required for a simulation involving a patient with depression, whilst fluent physical examination may be the focus of a station with a patient suffering from abdominal pain and associated emesis. The types of assessments and what they are specifically used to assess are determined throughout ‘blueprinting’. This process involves synthesising a master blueprint with a generalised overview of the entire curriculum; it is initially produced, highlighting the various aspects of the curriculum to be assessed as well as the type of assessment to be used [10].

Van Der Vleuten proposed a formula combining multiple elements that should be taken into account during the synthesis procedure and the use of an assessment method [24]. This set of parameters is used in conjunction to ensure that the proposed assessment is appropriate for what is intended to be tested; these include ‘reliability’, ‘validity’, ‘educational impact’, ‘acceptability’ and ‘cost’. The utility of an assessment is the product of each of these individual components resulting in the following formula: utility = reliability × validity × educational impact × acceptability × cost [25]. Over time, slight variations of the utility index have arisen, taking into account other additional factors such as feasibility [26].

Reliability of the OSCE

‘Reliability’ is typically defined as the reproducibility of assessment outcomes [27]. Being largely dependent on multiple factors including the type of assessment used, modes of quantifying reliability differ. In the context of written examinations, reliability is contingent on the concept of internal consistency, which is estimated by the use of either the Kuder-Richardson formula [28] or the Cronbach alpha coefficient [29]; these formulae are classically derived from the test-retest concept [27].

The OSCE is an examination involving multiple stations with standardised tasks and simulations of patients. Candidates are expected to progress through these stations, simultaneously receiving standardised marking off of a checklist with an additional global performance score [30]. Therefore, alternative statistical techniques are required to elicit the reliability of a clinical-based examination. OSCE performance ratings are susceptible to both internal and external errors, rendering them less reliable on the whole. Internal errors tend to include human-based factors such as differences in both a student’s motivation and interest in the examination [31], whereas external sources of error may span from the student’s gender to the ethnicity of the standardised patient as well as other examiner-centred influences [32,33]. Due to the nature and design of the assessment, various changes can be used to improve upon its reliability as an assessment tool.

Focusing specifically on examiners, quantifying reliability is based on ratings provided during the assessment, and any irreproducibility of students’ marks is usually due to discrepancies not only in the consistency of the same examiner but also, more importantly, between examiners (‘hawks’ or ‘doves’). This method of quantifying reliability between assessors is achieved by various statistical analytical techniques [27]; examples in the literature have included the employment of the Kappa coefficient, which has been shown to be of benefit in the estimation of inter-rater reliability [34].

However, beyond the scope of examiners, different sources of unreliability may be mitigated through more thorough analysis. Based on generalisability theory, previous analyses have shown that variance from multiple aspects of the assessment such as the student, the examiner and the items within the assessment can be calculated [35]. This is done with the use of modified variance component analyses, which take into account all the relevant factors that influence the result. This provides a more indicative reflection of the reliability of the assessment. In addition to this, it possesses the capacity to estimate reliability with multiple observations of clinical performance as well as the number of stations required to achieve an adequate level of reliability. To counteract the potential risk of subjectivity in candidates’ ratings, the placement of multiple assessors may be required at individual stations to reduce the error that may arise [36]. Reliability of an OSCE however requires the use of multiple stations [36], and this has been shown in the literature to be of more benefit than the use of multiple assessors; this has to be considered alongside the potential increase in costs that may arise from the recruitment of further simulated patients as well as the space required to carry out the assessment.

Validity of the OSCE

A second important aspect to be considered in the utility and quality of an assessment is its ‘validity’. In the context of assessments, validity often refers to whether a specific tool possesses the accuracy to actually measure what it has been designed to or intends to measure [37]. Another concept that is to be taken into account with regard to validity refers to the ability of the assessment to provide information that is also deemed appropriate for its intended purpose [38].

When focusing on the validity of an assessment, there are multiple major threats to consider [39]. However, the two most notable were proposed by Messick in 1989 [40]: construct under-representation (CU) and construct-irrelevant variance (CIV). Construct under-representation typically refers to the biased sampling of topics within an examination from the curriculum in such a fashion that no widespread coverage over the entirety of learning is achieved. Construct-irrelevant variance on the other hand refers to the incorporation of uncontrolled variables that possess the capacity to adversely distort the assessment outcomes. During assessment design, the effects of these sources of invalidity are typically accounted for, and measures are put in place to minimise their influence; blueprinting has been used as a technique in the past to prevent the effects of both CU and CIV [22].

As performance examinations such as the OSCE involve simulated patients and are simulations of real-world scenarios, certain issues with regard to validity arise. In the assessment of domains of performance, OSCEs are still artificial assessments. Students tend to focus on aspects such as the marking checklist for common scenarios to ensure higher marks are obtained during the assessment process [41], which resulted in the later incorporation of a globalised performance score. Due to the assessment’s design being primarily focused on a fixed number of stations demanding a certain level of performance with simulated cases, assessment scores are not necessarily reflections of how one is likely to behave in a clinical situation [27].

There are various CU and CIV-related factors that can result in a potential compromise of the validity of an OSCE. In relation to the design of a formative OSCE with only a few stations, construct under-representation poses the greatest risk to the validity of the assessment. The use of smaller numbers of clinical stations or smaller numbers of independent assessors impairs the validity of the assessment [27]. Each of these are issues with OSCE design that can arise especially in the context of a mock examination, and counter-measures have been documented within the literature. The work of Van Der Vleuten and Swanson [42] has demonstrated that to counteract risks to the validity of the OSCE, a recommendation of 12 simulated stations lasting a total of 20 minutes each is likely to be required. Low reliability that arises from using a few scenarios within the OSCE has a direct negative influence on the validity of the assessment [40]. Another critical point for potential concern may also include inadequate training being provided to simulated patients prior to assessment; disruption to the linearity of the assessment process between candidates results in students experiencing different clinical scenarios altogether. To ensure continuous provision of high-quality assessment, standardised monitoring is likely to be of benefit to ensure continuous correct portrayal of clinical cases and standardisation.

Educational impact of the OSCE

The educational impact of a proposed assessment varies depending on the aim behind its use. Summative assessments act as a metric to determine whether one has met the required level of both knowledge and skills to progress to the next stage of academic studies [2]. In the case of a formative assessment, the intention of the OSCE was to act as an opportunity for first-year students to direct their future learning [24]. Within this context, as opposed to an ‘assessment of learning’ of the curriculum, the focus was to act as an ‘assessment for learning’.

Criticisms of the OSCE have arisen in the past due to students often learning checklists for stations as well as focusing on common scenarios that they predict will appear in the examination [43]. This can lead to difficulty for faculty as they aim to drive students towards concentrating on the entirety of the curriculum. Despite these struggles, OSCEs have been shown to steer students’ behaviours with regard to their own learning [44]. The application of theoretical knowledge can only be achieved by candidates with the supplementary skill set required in a clinical setting; thus, acquiring skills in both communications and physical examination is necessary. After encouraging students to focus on the entirety of the curriculum, the OSCE results in motivation to focus on obtaining the necessary skills in more authentic learning environments in preparation for the assessment itself [45]. To ensure success in the examination, students partake in ward-focused learning activities such as clerking for a patient or performing a specialised physical examination [46].

Any feedback received from a formative OSCE could be used by students to identify areas of deficit within communication skills or allow them to continuously practice a physical examination or any specific steps missed or struggled with; tutor profiles with regard to assessors of stations have also shown to be an influential factor in the quality of feedback received. Findings have demonstrated that generalist assessors are preferred to specialists as students find the feedback provided to be more focused on the learner [47]. A caveat exists with regard to the provision of feedback in formative OSCEs. Although with the introduction of modern techniques to ensure criticism is delivered in a constructive manner, the processing of feedback at times may be difficult, resulting in impairment of a student’s self-efficacy and reduced morale [48]. Confidence in personal capabilities within performance-based examinations correlates to performance scores [49]. This can be especially challenging for students who are continuing to adjust to the difficulty of undergraduate medical studies after recently starting medical school.

Acceptability of the OSCE

The acceptability of utilising an assessment tool is dependent on the perception and understanding of its use held by both students and faculty [50]. Since its introduction as an assessment tool, the OSCE has allowed for adequate assessment of clinical competency in line with requirements set by regulatory bodies [5]. It has also been instrumental in providing feedback on the curriculum after identifying any areas for improvement with the curricular design [51]. Although it has provided benefits and has become more widely used since its initial introduction, little has been discerned in the literature on the specific viewpoints students hold towards the OSCE as an examination tool [52]. In the literature available, a running theme of OSCE-induced anxiety has been noted with various healthcare professionals indicating major apprehension prior to undertaking the assessment [52]. However, even in the case of a first-year medical student with no prior experience with the assessment, no significant evidence has revealed any difference in the levels of stress experienced compared to one who has experienced the OSCE in the past [53]. Students have however admitted to finding formative OSCEs to be of overall benefit, and previous exposure to the assessment has been seen as beneficial to student confidence when undertaking final assessments at a later stage [54]. Globally, the idea of a formative OSCE is generally seen in a positive light. Certain measures such as the use of debriefing meetings following assessments and suitable feedback have been shown to generally improve the overall perception and subsequently the acceptability of the OSCE by students [55].

Cost of the OSCE

Little analysis has gone into the cost implications of the OSCE as an assessment. Estimates have been formally published; however, multiple factors influence the overall cost of the examination tool. Considerations stem largely from the number of stations to be used within the OSCE. Naturally, the inclusion of greater numbers of stations results in increased costs due to secondary factors; a requirement for a greater number of simulated patients for communication skills cases, healthy volunteers for physical examinations and examiners is established with a larger number of stations. Other cost-baring concerns arise due to fixed costs of administrative staff required to carry out the process, although these costs are likely to remain fixed over consecutive assessments and are not amenable to realistic reduction [56]. Brown et al. [57] conducted a 15-station summative OSCE assessment for 185 students over the course of a two-day period at the University of Aberdeen as part of the undergraduate medical assessment process. An average of £355 pounds was required to run the assessment for each student, totalling near to £90,000 for the entirety of the assessment. Various cost reduction methods have been considered in the case of an OSCE assessment. Examples have included the employment of a sequential OSCE; however, these are of more benefit in longer summative cases. If students are to undergo a long course of stations over consecutive days, cost reduction can be achieved by sparing students from sitting the following days if a clear pass has been achieved on the first [58]. Other costs have to be considered, such as equipment and staff required for the sake of the assessment. The use of unpaid volunteers helps to reduce costs; however, they may be difficult to recruit. In addition to this, the replacement or reduction of consumables with viable options such as digitalised marking scales as opposed to paper checklists may help reduce the administrative costs required in the processing of assessment scores [59].

Cost-cutting practices are however enough to cause detriment to other aspects of the utility equation such as validity and reliability. Reductions in the number of stations have the potential to compromise the assessment as reliability is contingent on the number of stations within the assessment [60]. Other measures suggested within the literature such as the use of simulated patient assessors as opposed to assessors of medical background have proven to be unwise. Literature has revealed that the objectivity of the assessment is often affected as simulated patients tend to overscore medical students in the absence of a complete understanding of what is expected of them [61].

## Conclusions

Reflecting on this assessment type, it is clear that OSCEs have a well-established role in medical examinations. Despite certain concerns with regard to specific elements in the utility equation of the formative assessment, its use is to act as more of a learning point and help drive further learning. Certain changes to the design could be suggested; however, further implications may arise from this.

Alongside the appropriate design of the tool, the incorporation of appropriate statistical analyses is required to ensure the validity of the OSCE is of a high standard. The minimisation of both CU and CIV sources of error furtherly improves the validity of the formative OSCE as an assessment. Improving the reliability has additional benefits in the context of the assessment’s validity. However, these changes have to be balanced against another large factor within Van Der Vleuten’s utility equation: cost. The costs of a formative assessment are unlikely to be great in smaller examination circuits; accurate figures for individual components of the assessment may vary greatly and, moreover, are difficult to formally elicit. As the equipment and staff requirements for formative examinations are likely to be far less than those for summative assessment, it is presumed that these mock examinations consequentially are less costly. In addition to this, the involvement of medically trained educational staff belonging to the respective medical school is of benefit in encouraging that required competencies are met by students. Students are still likely to suffer from an element of anxiety within the OSCE; however, early exposure allows them to experience the examination’s format before their final summative. Although evidence is mixed with regard to whether it has any effect on future performances, the acceptability of such an assessment is influenced by students’ personal takes on it and understanding of its need within the examination process.

Overall, the formative OSCE is an assessment that, upon reflection, has benefits for undergraduate medical students; however, some concerns lie within its use. It is important to note that the primary aim of a formative OSCE is to both familiarise students with the testing format and improve learning; it has been shown to have a direct influence on learning behaviours within the educational processes of students. Evidence has outlined that it may not have any direct benefit on future performances with its use. Like other assessments before and after it, it possesses its own set of both advantages and disadvantages with potential means for improvement.
